# Cognitive impairments in patients with subacute coronavirus disease: Initial experiences in a post-coronavirus disease clinic

**DOI:** 10.3389/fnagi.2022.994331

**Published:** 2022-11-09

**Authors:** Jhin Goo Chang, Eun-Hye Ha, Wangjun Lee, Su Young Lee

**Affiliations:** ^1^Department of Psychiatry, Myongji Hospital, Hanyang University College of Medicine, Goyang-si, South Korea; ^2^Division of Pulmonary and Critical Care Medicine, Myongji Hospital, Hanyang University College of Medicine, Goyang-si, South Korea; ^3^Office of the Chief Executive Officer and Chairman, Myongji Hospital, Goyang-si, South Korea

**Keywords:** long COVID, cognitive function, subacute phase, cognitive sequelae, neurocognitive function test

## Abstract

**Background:**

A significant number of patients experience persistent cognitive impairment after coronavirus disease (COVID-19). This study aimed to investigate the cognitive function of patients in the subacute phase of COVID-19 and to identify the clinical factors associated with cognitive sequelae.

**Materials and methods:**

Data from patients who visited the psychiatric department of our post-COVID clinic between March and May 2022 were analyzed. The results of neuropsychiatric function tests, including the digit span forward (attention/processing speed) and backward (working memory) tests, the trail making test part A (attention/processing speed) and part B (executive functioning), and the Stroop word color interference test (executive functioning), as well as clinical data from 40 patients in the subacute phase of COVID-19 were analyzed. We calculated the frequency of impairments in each cognitive measure, defined as a z-score of ≤−1.5 standard deviations below measure-specific age- and sex-adjusted norms.

**Results:**

Of the participants, 72.5% (*n* = 29) had impairments in at least one cognitive domain. Impairment in executive function was the most frequent (64.9%), followed by impairments in processing speed/attention (52.5%) and working memory (42.5%). Age was inversely correlated with T scores in all cognitive function tests.

**Conclusion:**

Regular examination of cognitive function is needed, especially in elderly individuals, regardless of the subjective symptom manifestations.

## Introduction

Coronavirus disease (COVID-19) has caused more than 550 million confirmed cases of infection and over 6.3 million deaths worldwide by the end of June 2022 (WHO, 2022). A substantial proportion of individuals with COVID-19 have reported persistent symptoms beyond the acute illness, and these cases are referred to as “long COVID” (Taquet et al., 2021; O’Laughlin et al., 2022). According to recent literature, long COVID can be divided into two categories: (1) subacute or ongoing symptomatic COVID-19, which includes symptoms and abnormalities present from 4 to 12 weeks beyond acute COVID-19, and (2) chronic or post-COVID-19 syndrome, which includes symptoms and abnormalities persisting or present beyond 12 weeks of the onset of acute COVID-19 which are not attributable to alternative diagnoses (Nalbandian et al., 2021). After the acute phase of infection, fatigue and neurological and psychiatric symptoms are the most frequent symptoms during the chronic COVID-19 phase aside from respiratory, gastrointestinal, and cardiologic problems (Nasserie et al., 2021; Badenoch et al., 2022). Thus, the impact of COVID-19 varies among individuals, and long-term symptoms can have devastating effects (Praschan et al., 2021).

Brain fog, a term used to describe slow or sluggish thinking, is one of the most common symptoms reported by individuals who have survived COVID-19 (Heneka et al., 2020). Up to 80% of COVID-19 survivors have reported subjective cognitive decline from the acute to the chronic phase (Cirulli et al., 2020; Davis et al., 2021; Graham et al., 2021; Mazza et al., 2021; Guo et al., 2022a). Cognitive decline is often reported in the chronic phase and lasts for a long time (Ermis et al., 2021). In a cohort study including 273,000 COVID-19 survivors, neuropsychiatric symptoms were first reported after 90 days in a third of survivors, and many survivors who developed symptoms at an early stage also had symptoms that lasted up to 180 days (Taquet et al., 2021). In a systematic review that included studies reporting the results of objective neurocognitive tests, the onset of cognitive symptoms varied from the acute to the chronic phase of COVID-19 and persisted even 7 months after discharge (Crivelli et al., 2022). Therefore, when cognitive decline begins and how long it lasts are important concerns to be investigated.

In terms of cognitive domains, declines in attention, executive function, fluency, and memory have been commonly reported. Studies with patients in the acute phase of COVID-19 have reported declines in executive function, attention, memory, and verbal fluency (Groiss et al., 2020; Beaud et al., 2021; Hellmuth et al., 2021; Tolentino et al., 2021). Studies on post-COVID-19 patients also found cognitive deficits in verbal fluency, attention, executive function, and delayed memory (Davis et al., 2021; Ermis et al., 2021; Hosp et al., 2021; Miskowiak et al., 2021; Méndez et al., 2021). In a cohort study with 81,000 subjects including 12,000 confirmed COVID-19 cases, cognitive deficits were more evident in complex tasks requiring reasoning, planning, and problem solving as opposed to more basic working memory functions such as completing the digit span test (Hampshire et al., 2020). In a study focusing on long COVID, memory and executive function showed declines, but of the two domains, only the decline in memory remained significant after controlling for demographic variables (Guo et al., 2022b).

Several mechanisms underlying the neural damage caused by COVID-19 have been suggested, including direct invasion of SARS-CoV-2 into the brain or degenerative spread of the disease through olfactory pathways, abnormal ischemic or hemorrhagic events in the brain, neuroinflammation, and excessive immune responses (Douaud et al., 2022; Guo et al., 2022b). Importantly, this evidence was particularly strong in the presence of neurological symptoms (Helms et al., 2020; Kandemirli et al., 2020). Therefore, investigations of the neurocognitive decline associating with each phase of COVID-19, as well as demographic and clinical characteristics would be a cornerstone in revealing the pathophysiology of neurocognitive dysfunction caused by COVID-19.

An increasing number of studies have investigated the clinical correlates of COVID-19 infection (Davis et al., 2021; Douaud et al., 2022; Hampshire et al., 2022). Severe respiratory symptoms during the acute phase, older age, and hyposmia are associated with cognitive deficits. A recent long-COVID study with a community-based sample reported that fatigue/mixed symptoms during the initial illness predicted post-COVID cognitive symptoms, and different ongoing symptoms explained variance in individual cognitive tasks (Guo et al., 2022a,b).

In Korea, the peak of the COVID-19 pandemic occurred in March 2022 (WHO, 2022). Although an increasing number of patients complain of neurocognitive sequelae after the acute phase, reports of their incidence are insufficient. Myongji Hospital, which received the first Korean patient with COVID-19, is one of the representative hospitals specializing in infectious diseases and launched the “Purple Clinic,” the first for managing long-COVID in South Korea in March 2022. During the first 3 months of the Purple Clinic, 3,058 patients presented, and most patients were in the subacute phase of COVID-19. Therefore, we focused on identifying the characteristics and clinical correlates of cognitive impairment during the subacute phase of COVID-19. Many prior studies have reported cognitive impairments during the chronic/post-COVID phase. The subacute phase has been included in some studies but not in others as the phase classification for COVID-19 was still under discussion. We believe that investigation of the discrete subacute phase, or at least the early phase of chronic COVID-19, could demonstrate the transition of neurocognitive sequelae throughout long COVID.

## Materials and methods

### Participants

This study was approved by the Institutional Review Board of Myongji Hospital and was performed in accordance with the approved protocols and guidelines (MJH-2022-06-027). Data were collected from the Purple Clinic in Myongji Hospital, the first specialized clinic to care for patients with long COVID in Korea, from March to May 2022. During the first 3 months of the Purple Clinic, 3,058 patients presented, 59 of whom were referred for psychiatric consultation owing to their depressed mood, anxiety, or brain fog symptoms. Among the 59 patients, 40 patients in the subacute phase [between 28 and 90 days after the confirmation of COVID-19 using reverse transcription polymerase chain reaction (RT-PCR)] were finally included in the study.

### Subjective symptoms

In the Purple Clinic, all patients completed a subjective symptom checklist, which included 31 symptoms in eight categories: cardiopulmonary (coughing, productive sputum, shortness of breath, palpitations, chest pain, and edema), neurological (headache, dizziness, sleep disturbance, memory impairment, and tingling), gastrointestinal (abdominal discomfort, heartburn, abdominal pain, diarrhea, and nausea or vomiting), psychiatric (decreased attention, depression, and anxiety), general (fatigue, generalized weakness, and weight loss), ear-nose-throat (hyposmia and hypogeusia), eye (blurred vision and eye irritation), and others (hair loss and skin rash, dysmenorrhea, vaginal bleeding, bladder-related symptoms, foamy urine, and sexual dysfunction).

### Neuropsychological and cognitive function tests

Selected cognitive function tests [the digit span test, the trail making test (TMT), and the Stroop word color interference test], considering previous studies, were performed before visiting the psychiatric clinic (Biagianti et al., 2022). The tests provided data on three cognitive domains (attention/processing speed, working memory, and executive function) (Table 1). The time required to complete each test was recorded. We defined impairment in each measure as a z-score of ≤−1.5 standard deviations (SD) below the measure-specific age- and sex-adjusted norms. To reduce the use of the computationally cumbersome z-score, which can be positive or negative, we adopted the T-score system in the final analysis. The T-score is composed of a scale that ranges from 5 SD below the mean to 5 SD above the mean. Thus, for example, a raw score that fell exactly five SD below the mean would be equal to a T score of 0, a raw score that fell at the mean would be equal to a T of 50, and a raw score of five SD above the mean would be equal to a T of 100.

**TABLE 1:** Observed cognitive domains and respective neuropsychological tests.

Cognitive domain	Neuropsychological test
Attention/processing speed	Digit span forward
	Trail making test part A
Working memory	Digit span backward
Executive function	Trail making test part B
	Stroop word color interference test

Validated neuropsychological scales that measure mood [the Hospital Anxiety and Depression Scale, HADS (Snaith, 2003)], sleep quality [the Pittsburgh Sleep Quality Index, PSQI (Buysse et al., 1989)], distress after trauma [the Impact of Event Scale, IES (Weiss, 2007)] and fatigue severity [the Fatigue Severity Scale, FSS (Lee et al., 2013)] were also routinely used before visiting the psychiatric clinic to assess the referred patients’ symptoms on the day of presentation to the clinic. The HADS is a self-rating measure comprising seven items each for anxiety and depression. Each item is rated on a 4-point Likert scale ranging from 0 to 3, and the total score for depressive and anxiety symptoms ranges from 0 to 21 points each. The PSQI measures seven subdomains: subjective sleep quality, sleep latency, sleep time, usual sleep efficiency, sleep disturbance, use of sleeping pills, and daytime dysfunction. Each domain, rated on a 0–3-point scale, yields a total score ranging from 0 to 21, with higher scores indicating lower sleep quality. The FSS is a 9-item self-rating measure of the degree of fatigue experienced over the preceding week. Each item is rated from 1 to 7. The final FSS score is given by the average value divided by nine after adding the scores of each item. A higher score indicates higher fatigue. The IES is a 22-item self-report measure that assesses the subjective distress caused by traumatic events. Items are rated on a 5-point scale ranging from 0 to 4. The IES yields a total score ranging from 0 to 88, with higher scores indicating higher stress levels.

### Statistical analysis

We performed a descriptive analysis of the clinical variables. Spearman’s rho coefficient was calculated to determine the correlation between cognitive function and clinical characteristics. An additional multivariate regression analysis was performed, including age, HADS, PSQI, IES, and FSS results as independent variables. The Mann–Whitney test was used to assess the difference between the presence of subjective symptoms and cognitive function (T score). The threshold for statistical significance was α = 0.05, and all tests were two-tailed. Statistical analyses were performed using SPSS version 26 (SPSS Inc., Chicago, IL, USA).

## Results

### Sample characteristics

The demographic and clinical profiles of the participants are presented in Table 2. Forty patients in the subacute phase of COVID-19 were included in the study. The average age of the patients was 53.74 ± 16.46 years, and 51.95 ± 19.17 days had passed from SARS-CoV-2 infection confirmation using RT-PCR. The neuropsychiatric scales showed that the participants experienced significant levels of depression, anxiety, and sleep disturbances.

**TABLE 2:** Demographic and clinical characteristics of the participants (*n* = 40).

Characteristics	*n* = 40, Mean ± SD
**Sex**	
Female,% (n)	82.9% (33)
Age	53.74 ± 16.46
>60 years,% (n)	48.0% (19)
Days from the SARS-CoV-2 confirmation using RT-PCR	51.95 ± 19.17
Number of subjective symptoms	14.21 ± 5.87
HADS: Anxiety score	13.58 ± 4.92
HADS: Depression score	13.18 ± 3.90
FSS score	5.41 ± 0.23
IES score	41.08 ± 26.09
PSQI	12.47 ± 4.28

HADS, Hospital Anxiety and Depression Scale; FSS, Fatigue Severity Scale; IES, Impact of Event Scale; PSQI, Pittsburgh Sleep Quality Index; SD, standard deviation; RT-PCR, reverse transcription polymerase chain reaction.

### Frequency of subjective symptoms during the subacute phase of coronavirus disease

**FIGURE 1:**
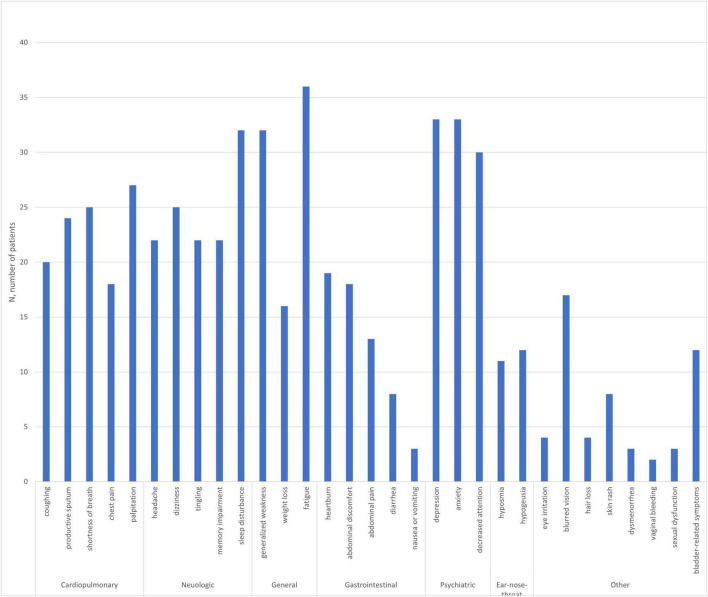
Subjective symptoms of the patients with subacute coronavirus disease referred to the psychiatric clinic (*n* = 40).

The average number of complained symptom was 14. Psychiatric, neurological, and general symptoms were also common (Table 2). The frequency of subjective symptoms among the participants is shown in Figure 1. In our previous report (Jung et al., 2022), the symptoms with higher prevalence in the post-acute (more than 4 weeks since the diagnosis of COVID-19) group were fatigue, decreased attention, depression, cognitive decline, blurred vision, hair loss, bladder symptoms, sexual dysfunction, and dysmenorrhea. Fatigue was the most common symptom among the patients.

### Cognitive function during the subacute phase of coronavirus disease

Neuropsychological test scores are presented in Table 3. The analysis indicated that 72.5% (*n* = 29) of the participants demonstrated scores of ≤−1.5 SD, compared with the adjusted norm, in at least one cognitive function test. Regarding each cognitive domain, impairments in executive function were the most frequent (64.9%, ≤−1.5 SD of the TMT-B or Stroop word color interference test results), followed by those in attention/processing speed (52.5%, ≤−1.5 SD of the digit span forward or TMT-A results) and working memory (42.5%, ≤−1.5 SD of the digit span backward results).

**TABLE 3:** Computerized neurocognitive test scores in the patients with subacute COVID-19.

Characteristics	Average direct score ± SD	T-score ± SD	Participants with ≤1.5 SD,% (n)
Digit span forward, *n* = 40	6.28 ± 1.38	45.18 ± 15.85	35.0 (14)
Digit span backward, *n* = 40	4.88 ± 1.33	45.65 ± 9.96	22.5 (9)
Trail making test part A (s), *n* = 38	38.97 ± 27.08	45.82 ± 16.13	34.2 (13)
Trail making test part B (s), *n* = 36	62.28 ± 35.82	48.89 ± 16.48	36.1 (13)
Stroop word color interference test (s), *n* = 38	41.58 ± 29.35	36.50 ± 12.18	63.2 (24)

s, second; SD, standard deviation; COVID-19, coronavirus disease.

### Correlates of cognitive function

Age was inversely correlated with T scores in all cognitive function tests (Table 4). According to multivariate regression analyses, age predicted lower cognitive function after adjustment for other clinical characteristics, including HADS, FSS, PSQI, and IES scores (Table 5).

**TABLE 4:** Correlation between the T scores in the cognitive tests and clinical characteristics.

	Attention/processing speed		Working memory	Executive function	
	Digit span forward	Trail making test part A	Digit span backward	Trail making test part B	Stroop word color interference test
Age	−0.638**	−0.750**	−0.639**	−0.745**	−0.852**
Days from the SARS-CoV-2 confirmation using RT-PCR	0.289	0.208	0.128	0.308	0.154
HADS: Anxiety score	–0.128	–0.226	–0.173	–0.230	–0.101
HADS: Depression score	–0.259	–0.211	–0.119	–0.123	–0.191
FSS score	0.020	0.085	0.177	0.142	0.024
IES score	0.164	0.086	–0.007	0.045	0.087
PSQI	–0.045	0.159	0.248	0.274	0.164
Number of subjective symptoms	0.077	0.076	–0.064	0.027	0.019

HADS, Hospital Anxiety and Depression Scale; FSS, Fatigue Severity Scale; IES, Impact of Event Scale; PSQI, Pittsburg Sleep Quality Index; RT-PCR, reverse transcription polymerase chain reaction. **p < 0.001.

**TABLE 5:** Linear regression results for cognitive function by age and other clinical characteristics.

Predictor	Cognitive task	*F*	*P*	*B*	*t*	Adjusted *R*^2^
Age	Digit span forward	4.830	<0.001	−0.598	−4.342	0.383
	Trail making test part A	4.683	0.003	−0.417	−3.267	0.387
	Digit span backward	6.932	<0.001	−0.374	−4.478	0.490
	Trail making test part B	8.485	<0.001	−0.670	−5.364	0.576
	Stroop word color interference	8.456	<0.001	−0.546	−5.853	0.561

Age, Hospital Anxiety and Depression Scale; Impact of Event Scale; Pittsburg Sleep Quality Index, and Fatigue Severity Scale results were entered as independent variables. Variables that showed significant results (*p* < 0.05) are presented in the table as predictors.

Regarding each subjective symptom (Table 6), patients with headaches had lower digit span backward scores than those without headaches (average ranking: 23.32 vs. 15.71, *p* = 0.039). Patients with subjective memory impairment and weight loss had lower TMT-A scores than those without subjective memory impairment (average ranking: 23.03 vs. 15.58, *p* = 0.036) and weight loss (average ranking: 22.20 vs. 14.30, *p* = 0.028). Furthermore, there was a trend level of difference in the TMT-A results between patients with and without hyposmia (21.27 vs. 13.64%, *p* = 0.051). When multiple linear regression was performed, including all 17 symptoms, no symptoms significantly predicted the results of the cognitive tasks. Note that there were 31 symptoms on the checklist, and 17 symptoms with at least 10 cases in each group (with or without symptoms) were included in the comparisons. Nausea/vomiting, diarrhea, eye symptoms, hair loss, dysmenorrhea, abnormal vaginal bleeding, and sexual dysfunction were excluded due to the small number of cases. On the other hand, depression, anxiety, insomnia, decreased attention, fatigue, and loss of energy were excluded because of the small number of cases without such symptoms (non-cases). This was an inevitable result because the study population was referred to a psychiatric clinic due to these symptoms. The effects of the psychiatric symptoms and fatigue on cognitive function were investigated by comparisons (Tables 4, 5).

**TABLE 6:** Comparisons of the cognitive test results across the subjective symptoms.

		Attention/processing speed		Working memory	Executive function	
		Digit span forward	Trail making test part A	Digit span backward	Trail making test part B	Stroop word color interference test
Palpitation	*Z*	–1.196	–0.783	–0.276	0.000	–0.424
	*p*	0.245	0.441	0.799	1.000	0.707
Shortness of breath	*Z*	–0.531	–0.559	–0.471	–0.436	–1.049
	*p*	0.613	0.582	0.654	0.668	0.337
Dizziness	*Z*	–0.546	–0.144	–0.766	–0.570	–0.086
	*p*	0.592	0.888	0.460	0.587	0.936
Sputum	*Z*	–0.305	–1.040	–0.320	–0.869	–0.247
	*p*	0.765	0.304	0.765	0.400	0.819
Headache	*Z*	–1.927	–0.723	−2.081*	–1.187	–1.402
	*p*	0.055	0.476	**0.039**	0.240	0.195
Tingling	*Z*	–1.827	–0.477	–1.838	–0.591	–0.592
	*p*	0.072	0.639	0.067	0.561	0.593
Memory impairment	*Z*	–0.314	−2.094*	–0.670	–1.076	–1.232
	*p*	0.769	**0.036**	0.510	0.287	0.257
Coughing	*Z*	–0.305	–1.040	–0.320	–0.869	–0.247
	*p*	0.765	0.304	0.765	0.400	0.819
Heartburn	*Z*	–0.156	–0.963	–0.156	–1.179	–0.307
	*p*	0.879	0.341	0.879	0.243	0.775
Abdominal discomfort	*Z*	–0.453	–0.244	–0.099	–0.480	–0.146
	*p*	0.667	0.822	0.923	0.636	0.892
Chest pain	*Z*	–0.795	–0.061	–0.524	–0.745	–1.005
	*p*	0.443	0.964	0.606	0.463	0.357
Weight loss	*Z*	–0.791	−2.188*	–0.819	–1.537	–0.983
	*p*	0.437	0.028	0.420	0.127	0.360
Abdominal pain	*Z*	–1.062	–0.534	–1.326	–0.702	–0.795
	*p*	0.303	0.610	0.195	0.489	0.460
Hypogeusia	*Z*	–0.782	–0.633	–0.061	–0.147	–0.309
	*p*	0.443	0.544	0.964	0.900	0.775
Hyposmia	*Z*	–1.368	−**1.967**	–1.272	–1.155	–0.873
	*p*	0.177	**0.051**	0.210	0.255	0.428
Blurred vision	*Z*	–1.395	–0.842	–0.863	–0.998	–1.290
	*p*	0.163	0.400	0.388	0.318	0.197
Bladder-related symptoms	*Z*	–1.499	–0.641	–0.199	–0.316	–1.19
	*p*	0.134	0.521	0.842	0.752	0.234

Bold values represent the *p* ≤ 0.051. *p < 0.05.

## Discussion

This study is the first in Korea to examine cognitive sequelae in patients in the subacute phase of COVID-19. The strength of this study is that cognitive functions were examined within a specific period, between 28 and 90 days after the confirmation of COVID-19, using objective cognitive tests. The examinations using objective cognitive tests showed that a significant number of patients had impairments in executive function and attention/processing speed. In particular, the older the patient, the more severe the cognitive impairment compared to age-adjusted norms. Routine inspection using objective neurocognitive tools is required for early detection, especially in elderly patients.

The results of our study are consistent with those of previous studies that investigated the prevalence of cognitive deficits in patients in the subacute phase. In particular, one study investigated cognitive function using the Montreal Cognitive Assessment in 53 hospitalized patients and 61.5% of patients had deficits in cognitive function, primarily in executive function, attention, language, and delayed recall (Ermis et al., 2021). Another study that conducted cognitive function tests at the 12th week of diagnosis with 130 patients discharged after treatment for COVID-19 reported that executive function and psychomotor coordination were impaired in 50–75% of patients (Mazza et al., 2021). All participants in our study had confirmed SARS-CoV-2 infection during the Omicron-variant era, and the severity of acute symptoms was relatively low. Our results suggest that observation of cognitive sequelae is needed even in patients who suffered from mild symptoms in the Omicron era and did not require hospitalization in the acute phase. Further, this argument is strengthened by a recent case-control study that reported significant cognitive decline and brain structural changes after SARS-CoV-2 infection regardless of hospitalization (Douaud et al., 2022).

There is now a large body of literature on neurocognitive sequelae associating with cognitive domains and clinical characteristics. With respect to cognitive domains, more evident impairments in higher cognitive functions were reported in a large cohort study of 12,689 individuals who were suspected to have COVID-19 (Groiss et al., 2020). This study did not specify the time since COVID-19 was confirmed, and the degree of severity of respiratory symptoms in the subjects varied. In a study focusing on 181 cases of long COVID, memory exhibited the only significant decline among the cognitive domains after controlling for age, sex, country, and education level (Guo et al., 2022b). In that study, there was a significant group difference in reaction time on the executive function test, but this dropped below significance after adjustment. In another study with 100 subjects visiting a Neuro-COVID-19 clinic, short-term memory and attention were the most commonly impaired domains (Davis et al., 2021). This study included 50 non-hospitalized SARS-CoV-2 laboratory-positive individuals and 50 laboratory-negative individuals. In our study, the most commonly impaired domain was executive function (Stroop word color interference and TMT-B), followed by attention/processing speed (digit span forward and TMT-A). We defined impairments in each measure as a z-score of ≤−1.5 SD below the measure-specific age- and sex-adjusted norms. However, education level was not controlled for, and there was no control group in our study. It is also necessary to consider that the tasks representing each cognitive domain differed by study. Otherwise, cognitive impairment in this study may be characteristic of subacute patients who experienced relatively milder symptoms during the Omicron era. The absence of a memory test in the battery of day-of-visit cognitive tests is a limitation of our study. Taken together, memory, executive function, and attention domains need to be investigated according to the phases and characteristics of subjects with COVID-19.

In terms of clinical characteristics, as patients aged, cognitive function declined more than the age-and sex-adjusted norms in all cognitive domains. Previous studies have also shown that cognitive decline in patients with post-COVID syndrome is more prominent in older patients (Kouzuki et al., 2021; Badenoch et al., 2022; Douaud et al., 2022). Interestingly, in our study, significance was maintained after adjusting for the severity of psychiatric symptoms and fatigue. Furthermore, severity of psychiatric symptoms was not related to age (Supplementary Table 1). This suggests that cognitive decline could be a sequela of the viral disease, not merely a symptom related to fatigue, mood, or anxiety.

Several mechanisms of cognitive decline after COVID-19 have been suggested, and structural and functional imaging studies are accumulating (Hosp et al., 2021; Aoun Sebaiti et al., 2022); however, many aspects remain unknown. Although the purpose of our study was not to elucidate the underlying mechanisms, our results provide some clues. Our study found that the frequency of executive function decline was common in the subacute phase and was not associated with other subjective ongoing symptoms. In contrast, attention, processing speed, and working memory deteriorated more in participants who reported subjective memory loss (TMT-A), weight loss (TMT-A), and headache (digit span backward) than in participants who did not. The gray matter thickness and tissue contrast in the orbitofrontal cortex, which is associated with executive function, were significantly reduced in COVID-19 survivors compared to controls, and this significance was maintained after excluding patients hospitalized for severe symptoms (Douaud et al., 2022). In a previous study with immune markers, an increased systemic inflammation index in the acute phase predicted further cognitive decline in processing speed and coordination but did not predict declines in executive function (Mazza et al., 2021). These results suggest that deterioration of executive function might be a symptom independent of the severity of systemic inflammation. Further studies with the same group after the systemic symptoms disappeared are needed to clarify this.

Headache is one of the most common neurological symptoms among the general population. Headache was correlated with the results of the digit span backward test, which showed the smallest percentage of decline in our study, as well as in a prior large cohort study (Groiss et al., 2020). In another study, headache severity was associated with performance on the word recognition test, category fluency, and pictorial associative memory (Guo et al., 2022b). Therefore, whether this correlation is disease specific or a more general manifestation needs to be investigated in studies with control groups. In addition, factor analysis studies to identify the features of long COVID are important.

Interestingly, participants with hyposmia tended to show decreased performance on the TMT-A at the trend level (*p* = 0.051). In addition, there were no differences in age or other psychiatric symptom scale results between the hyposmia and non-hyposmia groups (Supplementary Table 2). Decreased performance on the TMT-A, which reflects a concentration problem, has been frequently found in neuroinflammatory conditions, such as chronic fatigue and chemobrain syndrome (Aoun Sebaiti et al., 2022). Consistent with this result, COVID-19-related hyposmia has recently been shown to be associated with viral persistence and neuroinflammation (de Melo et al., 2021). The presence of hyposmia in the subacute phase may be attributed to ongoing neuroinflammation, which further affects cognitive function.

This study has several limitations. First, our results cannot be generalized to all patients with subacute COVID-19 because the data were obtained from patients who had been referred to a psychiatric clinic. However, depression, anxiety, and other psychiatric symptom severities were not associated with cognitive functional outcomes (Tables 4, 5). Second, we could not check all cognitive domains, including memory function, because the tests were conducted on the day of presentation for patients who visited from afar owing to their long-COVID. Third, although the checklist contained a total of 31 symptoms covering all systems, we could not compare cognitive function based on all subjective symptoms owing to the small number of cases or non-cases in some symptoms. In addition, the difference in cognitive function by clinical symptoms was not significant in multiple linear regression; therefore, these results need to be taken as exploratory demonstrations for future research. A large-scale longitudinal study is required to determine the cognitive trajectory of COVID-19 patients. Fourth, it is difficult to establish the extent to which cognitive change is due to COVID-19 infection specifically, or other factors related to the pandemic period, which has been one of the most stressful conditions for many people, regardless of infection status. We showed that cognitive decline was not correlated with current psychiatric symptoms, but a comparison with a non-infected control group would be preferable.

Nevertheless, this study has several strengths. First, this is the first study to report the objective cognitive sequelae of patients with COVID-19 in South Korea and showed that the characteristics were consistent with results from other countries. Second, this study demonstrated cognitive function in patients in the subacute phase of COVID-19 and suggested that the cognitive sequelae of COVID-19 could start before the chronic phase, especially among older patients. Third, this study showed a separate cognitive decline that was not fully explained by psychiatric symptoms and explored the relationship between cognitive sequelae and the systemic symptoms of COVID-19.

## Data availability statement

The original contributions presented in the study are included in the article/Supplementary material, further inquiries can be directed to the corresponding author.

## Ethics statement

The studies involving human participants were reviewed and approved by Institutional Review Board (IRB) of Myongji Hospital. The ethics committee waived the requirement of written informed consent for participation.

## Author contributions

SL and WL: conceptualization. JC: data curation, validation, visualization, and writing—original draft. SL: formal analysis and supervision. All authors contributed to writing—review and editing, investigation, and methodology.
