# Supplementary material for: Cognitive impairments in patients with subacute coronavirus disease: Initial experiences in a post-coronavirus disease clinic
Source: Front Aging Neurosci. 2022 Nov 9;14:994331. doi: 10.3389/fnagi.2022.994331 (PMC9681802; doi:10.3389/fnagi.2022.994331)
Supplement: Supplementary file 1 [file Table_1.DOCX]

|  | **HAD-Anxiety score** | **HAD-Depression score** | **FSS score** | **IES score** | **PSQI** |
| --- | --- | --- | --- | --- | --- |
| **Age** | .175 | .079 | -.103 | -.131 | -.183 |
| **Days from SARS-CoV-2 infection** | .063 | .147 | .111 | .290 | .017 |
| **Number of symptoms** | .353* | .457** | .353* | .739** | .513** |
| **Number of symptoms**  **(Without neuropsychological symptoms)** | .268 | .361* | .258 | .690** | .515** |
| HAD = hospital anxiety depression scale; FSS = fatigue severity scale; IES = impact of event scale; PSQI = Pittsburg sleep quality index; **p* < 0.05 ; ***p* < 0.01 | | | | | |

**Supplemental Table 1.** Correlation between the neuropsychological symptoms and clinical characteristics
