# Supplementary material for: Cognitive impairments in patients with subacute coronavirus disease: Initial experiences in a post-coronavirus disease clinic
Source: Front Aging Neurosci. 2022 Nov 9;14:994331. doi: 10.3389/fnagi.2022.994331 (PMC9681802; doi:10.3389/fnagi.2022.994331)
Supplement: Supplementary file 2 [file Table_2.DOCX]

**Supplemental Table 2.** Distribution of the demographic and clinical characteristics in the hyposmia and non-hyposmia groups

|  | Hyposmia  (n = 11) | Non-hyposmia  (n = 27) | z | *p* |
| --- | --- | --- | --- | --- |
| Age | 20.77 | 19.70 | -.265 | .791 |
| Days from the SARS-CoV-2 confirmation using RT-PCR | 25.18 | 17.96 | -1.780 | .075 |
| HAD-Anxiety score | 22.32 | 18.35 | -1.001 | .317 |
| HAD-Depression score | 24.27 | 17.56 | -1.696 | .090 |
| FSS score | 18.09 | 20.07 | -.500 | .617 |
| IES score | 22.09 | 18.44 | -.918 | .359 |
| PSQI | 22.82 | 18.15 | -1.181 | .238 |
| HAD = hospital anxiety depression scale; FSS = fatigue severity scale; IES = impact of event scale; PSQI = Pittsburg sleep quality index | | | | |
